# Distribution of Heavy Metals in the Soils Associated with the Commonly Used Pesticides in Cotton Fields

**DOI:** 10.1155/2016/7575239

**Published:** 2016-03-09

**Authors:** Saadia Rashid Tariq, Musharaf Shafiq, Ghayoor Abbas Chotana

**Affiliations:** ^1^Department of Chemistry, Lahore College for Women University, Lahore 54000, Pakistan; ^2^Department of Chemistry, Syed Babar Ali School of Science & Engineering, LUMS, Lahore 54792, Pakistan

## Abstract

Agricultural soils contain both heavy metals and pesticides originating from various agricultural practices. It is quite important to study the relationships between these two classes of compounds. To accomplish this, 52 soil samples were collected from cotton fields and analyzed for their metal contents (Ni, Cu, Co, Pb, Cr, and Cd) and levels of most commonly used pesticides (imidacloprid, acetamiprid, and emamectin). FAAS was used for metal estimation and the pesticides were determined by HPLC equipped with UV detector. The results of the study revealed slightly enhanced levels of Ni and Cd in these samples while the rest of the metals were present within tolerable range. Acetamiprid residues in soil were strongly positively correlated with Cu and negatively correlated with Cr. Similarly, imidacloprid in soil was negatively correlated with Ni. Thus it was evidenced that Cu stabilizes acetamiprid while Cr and Ni facilitate the degradation of acetamiprid and imidacloprid in the soil.

## 1. Introduction

Pesticides and heavy metals are the most hazardous contaminants of agricultural soils. In fact, the agricultural sustainability has long been associated with the use of a broad spectrum of pesticides that control the disease causing pests and crop destroying insects [1]. According to an estimate, in Pakistan alone about 20379 metric tons of pesticides is used annually with about 75% of these being used only for cotton crops [2]. That is why cotton fields have been found to be more contaminated than other modes of land use [3]. A significant portion of these pesticides (about 19%) find their way into the soil through spray drifts, or as wash-off from treated foliage [4]. From the soil, these pesticide residues may get directly vaporized along with the evaporating soil moisture or are leaked into the ground and surface waters through leaching or run-off [5–8]. The situation is more aggravated if a mixture of pesticides is present in the soil because they render the remediation process to be more difficult [9]. The pesticides cause various diseases among the exposed individuals such as cancers of various types and impairment of kidneys and thyroid glands [10]. Moreover, these pesticides have also proved to be detrimental to nontarget, beneficial soil microorganisms. That is why the determination of levels of pesticides in soil, groundwater, and crops has always been the subject of interest [11–14].

Pesticide degradation is an important phenomenon that helps in remediating the contaminated soils and water bodies. It is shown to be assisted by a number of factors such as moisture content of soil, soil texture, and soil mineral contents [9]. The presence of metals in the soil has also recently been shown to affect the degradation of pesticides [15]. These metals either are added to the soil along with pesticides or originate from the long-term application of wastewater and animal manures to agricultural soils [16, 17]. In fact metals affect the pesticide degradation by catalyzing the photolysis/hydrolysis of pesticides or influencing the activity of microorganisms [18]. Similarly, complexation interactions between pesticides and metals may lead to a decrease in the rate of degradation of pesticides [19].

Tian et al. studied the influence of bimetallic Ni/Fe nanoparticles on the degradation of DDT in aqueous solutions at room temperature [20]. Liu et al. observed that coexisting Cu ions in the soil influence the degradation of cypermethrin and cyhalothrin by affecting the activity of microorganisms [21]. Complexation interactions between imazapyr pesticide and metal ions were found to decrease the imazapyr photolysis [22]. It has also been found that selected metal ions having paramagnetic property could inhibit the aquatic photodegradation of some pesticides with humic acids [23].

The present study focuses on tracing the correlation patterns among the levels of coexistent metals and pesticides in the soil. The correlation model thus developed between pesticide and heavy metals will be useful for monitoring the pesticides in soil, due to the measurement of heavy metals being more available. So, the study may provide the basis for the management of soils contaminated with pesticides subsequent to successful monitoring.

## 2. Materials and Methods

### 2.1. Quality Control and Quality Assurance

High quality Pyrex glassware was used during the present study which was thoroughly cleaned with chromic mixture, 5% detergent solution, and finally distilled water. All the volumetric apparatus employed for measurements, sample processing, and dilution was calibrated before use. In order to deliver the small volume (i.e., <1.0 mL) of stock standard solutions, pipettes of 1.00 mL capacity were used.

Double distilled water was used for the preparation of blanks, standards, and reagents. Analytical grade reagents with a 99.99% certified purity were procured from E-Merck (Germany) and used for quantification. The stock standard solutions of selected metals (1000 mg/L) as provided by E-Merck (Germany) were utilized for the preparation of working standards after appropriate dilution with DDW. The inconsistencies in measurement likely to arise from the use of highly concentrated stock solutions were avoided by the use of an intermediate working standard of 100 mg/L concentration. HPLC grade chemicals provided by E-Merck Germany were used for the extraction of pesticides, sample cleanup, and mobile phase formation. The analytical standards of three pesticides were obtained from Riedel-deHaen (Germany). The chances of any photodegradation of reagents were avoided by storing them in the dark.

The levels of selected trace metals, that is, Co, Pb, Cr, Ni, Cu, and Cd, were determined by using HITACHI AAS Z-5000 spectrophotometer. The calibration curve method was used to quantify the results. At least six standards were run covering the absorption range of samples. The precision of quantitative results was ensured by analyzing the triplicate samples.

The residues of three selected pesticides, that is, imidacloprid, emamectin, and acetamiprid, were determined by using the HPLC System of Waters 1500. The detector used for the present investigation was Waters-2487, a dual wavelength absorbance detector, designed especially for HPLC applications.

The extracted and cleaned pesticide residue samples were injected into the chromatographic system equipped with C-8 column at a rate of 1 mL/min. The eluent used was a 3 : 2 mixture of water and acetonitrile. At the retention times of 4.93 minutes, the signals for imidacloprid, emamectin, and acetamiprid were recorded by UV detector at specified wavelengths of 270 nm, 245 nm, and 254 nm, respectively.

### 2.2. Sampling

For the present investigation, a total of 52 top soil (1–15 cm) samples were collected from ten different fields of Pakpattan, Pakistan. A 300 g portion of each of the soil samples was taken in zip mouthed polyethylene bags with the help of soil auger. These samples were properly labeled and immediately transferred to lab, where they were stored at 4°C until analysis. The electrical conductivity and pH of the samples were determined by immersion probe technique while moisture content, chloride, nitrate, and sulphate were determined by appropriate standard methods [24]. All samples were treated and analyzed at the laboratory of Department of Chemistry, Lahore College for Women University, Lahore. In order to get the reference data, the background soil samples were collected from remotely located agricultural soil with sandy loam texture, where no pesticides were used previously. These background soil samples were also processed quite similar to the samples from the fields.

### 2.3. Description of Study Area

**Figure 1:**
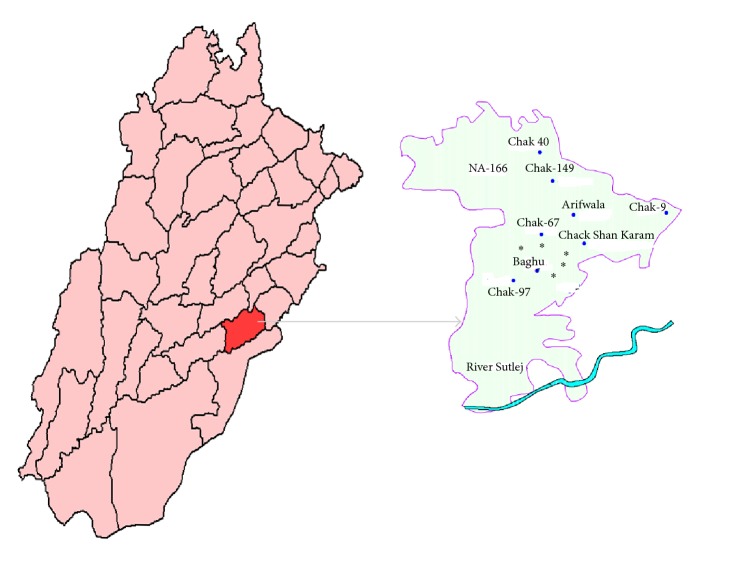
Location map of sampling sites^*∗*^.

The district Pakpattan is one of the major cotton growing areas in Pakistan. It covers an area of about 3,084 km^2^ with a population density of 417 persons per sq.km. A highly fertile land and a climate that is most suitable for sustaining the crops are the main features of the area. The city is bounded by river Satluj and Bias that constitute the main source for irrigation. Therefore, 88% of the area is comprised of cultivated land. Soil texture here varies from silt loam to clay loam and average annual rainfall is 200–300 mm. The temperature ranges from 6.6°C in winter to 41.7°C during the summer. The main crops grown here include wheat, rice, cotton, maize (corn), and sugar cane. In addition, about 507 agriculture based industrial units are present in the district that include fertilizer, cotton pressing and ginning, oil mills, flour mills, rice mills, poultry feed, textile weaving, and sugar mills. The exports from the area consist principally of cotton, wheat, and oilseeds. To accomplish the present study, a total of fifty-two samples were obtained from cotton growing areas of Pakpattan. The sampling sites location is shown in Figure 1.

### 2.4. Determination of Physicochemical Parameters

The collected soil samples were ground, homogenized, and subsequently sieved through a brass sieve with pore size of 850 *µ*m in accordance with international standards for soil analysis [24]. The water extract of soil samples was prepared by stirring a 1 : 10 soil-water mixture for five minutes and allowing equilibration for thirty minutes. The filtrate thus obtained was used for the determination of physicochemical parameters such as chlorides and nitrates sulphates [25]. In order to determine the soil bulk density, undisturbed cores of dimensions 0.05 × 0.072 m were drawn from 5 to 10 cm depth of soil [26].

The pH of all the soil samples was determined by using a Inolab-720, portable pH meter, precalibrated by using standard buffer solutions of pH 4.0, 7.0, and 9.0. The conductivity of water extract of soil samples was determined by using WTW, Inolab-720 conductivity meter.

The chloride contents of the soil samples were determined gravimetrically by using AgNO_3_ as precipitant [27]. In order to determine the sulphate ions, a 10.0 mL portion of water extract of soil samples was stirred for five minutes with HCl and glycerol solutions along with approximately 0.03 g of BaCl_2_. Immediately, after stirring, the solution was poured into a quartz cell with an optical path of 1.0 cm and the absorbance was measured at 420 nm on a UV spectrophotometer. A series of calibration standards between concentrations of 0.5 and 5 mg SO_4_^2−^/L were prepared and used for drawing calibration curve [24].

Nitrate present in the soil samples was determined by using the sodium salicylate method. Here 10.0 mL portions of water extract of each of the soil samples were evaporated with 1.0 mL of sodium salicylate and cooled to room temperature. Subsequently 1.0 mL of concentrated H_2_SO_4_ was added to dehumidify the entire residue and allowed to stand for 10 minutes. It was then quantitatively transferred to a 50 mL volumetric flask, added with 7.0 mL of NaOH, and adjusted the volume to 50 mL with distilled water. After 10 minutes, the absorbance was measured at 410 nm against the blank prepared in the same way. Calibration curve was constructed by using the standard KNO_3_ solutions in the concentration range of 2–20 mg/L [28]. The organic carbon contents of the soil samples were determined by partial oxidation method that involved the titration of soil samples against 1 N (NH_4_)_2_Fe(SO_4_)_2_ · 6H_2_O solution by using diphenylamine indicator [29].

*Pesticide Analysis by HPLC*. The residues of selected pesticides, that is, imidacloprid, acetamiprid, and emamectin, were extracted from soil samples by using methanol as solvent. The extraction procedure adopted involved the shaking of the appropriate amount of soil samples with 100 mL of methanol on an orbital shaker for 30 minutes. The residual cake was extracted twice with 100 mL methanol. The methanol extracts were combined, filtered, and then concentrated under reduced pressure to dryness at 40°C.

*Sample Cleanup*. The contaminated sample extracts cause the loss of detector sensitivity, shorten the life time of column, produce extraneous peaks, and deteriorate peak resolution and column efficiency [27]. Therefore, the extract was cleaned by using a Florasil packed column. Briefly, about 20 g of Florasil was added to a preconditioned chromatographic column between two layers of anhydrous sodium sulphate (1 to 2 cm deep). The concentrated extract was loaded onto the top of the column. The column was drained with acetonitrile until the sodium sulphate layer was exposed. It was then eluted with mobile phase at a drip rate of about 5 mL/min. Mobile phase for HPLC analysis was prepared by mixing 0.01% of acetic acid and acetonitrile in 60 : 40 ratio. The eluate was concentrated and residue was redissolved in 5 mL of acetonitrile. This cleaned extract was used for pesticide analysis by HPLC.

### 2.5. Metal Analysis by AAS

Wet acid digestion method was used for the analysis of metals present in soil samples. Briefly, 1.0 g portions of air-dried and sieved soil samples were added with 15 mL of 1 : 1 conc. HNO_3_ and refluxed without boiling at 95°C for about 10–15 minutes. It was further added with 3 mL of concentrated HNO_3_ and heating was continued for about 30 minutes. Subsequently, the contents were cooled to room temperature and added with about 5 mL of 30% H_2_O_2_ in aliquots. Digestion was continued until effervescence subsided. Then, a 15 mL portion of concentrated HCl was added and contents were cooled, filtered through a sintered glass crucible, and finally diluted up to 50 mL with distilled water [30]. This acid extract was aspirated directly onto AAS for metal estimation under optimum analytical conditions as given in Table 1.

### 2.6. Statistical Analysis of Data

The data set obtained for various physicochemical parameters, metals, and pesticide residues in the representative soil samples was treated statistically by both univariate and multivariate statistical analyses. The univariate statistical analysis involved the evaluation of basic statistical parameters like mean, standard deviation, standard error, kurtosis and skewness, and the linear correlation coefficient matrix. Correlation coefficient matrix furnished the correlation patterns among various metal pairs, physicochemical parameters, and pesticides in the soil media. Multiple correlation matrix was also developed in terms of metal-to-pesticide, metal-to-physicochemical parameters, and pesticide-to-physicochemical parameters to determine the dependence of various parameters upon each other. The multivariate statistical analysis in terms of cluster analysis was performed to get an insight into the clustering behavior of various parameters on mass scale by using Statistica software [31].

## 3. Results and Discussion

### 3.1. Distribution of Various Parameters of Soil Samples

Physical properties of the soil influence the mobility and pathways of nutrients and pollutants within the soil. Similarly, the accumulation of the xenobiotics (foreign chemicals) in the soil is governed by a number of physicochemical parameters of soil such as its pH, conductance, and the amount of inorganic ions (Cl^−^, NO_3_^−^, SO_4_^2−^, etc.). It is therefore necessary to evaluate these physicochemical parameters and determine their mutual relationship so that a sustainable agriculture may be maintained.

The basic statistics for the physicochemical parameters, metal levels, and pesticide concentrations in fifty-two soil samples collected from ten fields of Pakpattan, Pakistan, are enlisted in Table 2. The soil samples were grayish in colour and the texture of all the soil samples was sandy loam. The bulk density of soil samples was found to range from 1.53 to 1.61 Mgm^−3^ with a mean value standing at 1.568 Mgm^−3^. pH, another important parameter for the agricultural soil, was found to be present at narrow range of 7.6–8.5, with a mean value of 8.1. Soils with these pH values exhibit higher availability of metals such as Mg, Ca, and K while some other metals such as Fe, Zn, and Cu are less available at this pH [32]. It is also known that, under alkaline conditions, the adsorption of pesticides takes place to a lesser extent, and thus they degrade easily [33–35].

The organic matter content of soil is regarded as a major factor controlling the sorption, transport, and transformation of pesticides [36]. For the present soil samples, it was found to vary from 1.29 to 11.39% with a mean value of 4.616%. An organic carbon content of >5% may enhance the sorption of pesticides in soil depending on the nature of pesticides and organic matter; this in turn reduces the leaching and transport properties of pesticides [37]. In case of present study, the mean organic carbon content of soils was found below 5%, but slightly higher than background levels. Thus in these soils, the extent of sorption of pesticides to the soil may not be significant, but this may be evidenced clearly on the basis of a more comprehensive study based on chemometrics.

The ionic strength of these soil samples is represented by a mean conductivity value of 1412 *µ*S/cm, signifying that a larger portion of solubilized cations and anions are present in the water extracts of soil samples, which were most likely derived from the fertilizers and manure used to restore the fertility of soil. It was further confirmed by the presence of high concentration of anions such as chloride, nitrate, and sulphate in the soil samples.

The mean chloride, nitrate, and sulphate levels recorded for these soil samples were 12.9, 35.01, and 47.1 mg/g, respectively, with their sample variances of 3.77 × 10^7^, 1.178 × 10^8^, and 4.66 × 10^8^, respectively. The order of mean levels of these anions thus remained: SO_4_^2−^ > NO_3_^−^ > Cl^−^. Large values for standard deviation projected a large spread of data set around the mean value.

The excessive sulphate concentration in the soil originates from the use of zinc sulphate and urea in addition to decayed vegetable matter, compost, and manure used to normalize the soil pH. The enhanced levels of nitrate in the soil as compared to background soil pointed towards an excessive use of nitrate fertilizers such as nitrates of sodium, potassium, calcium, and ammonium in fields.

**Figure 2:**
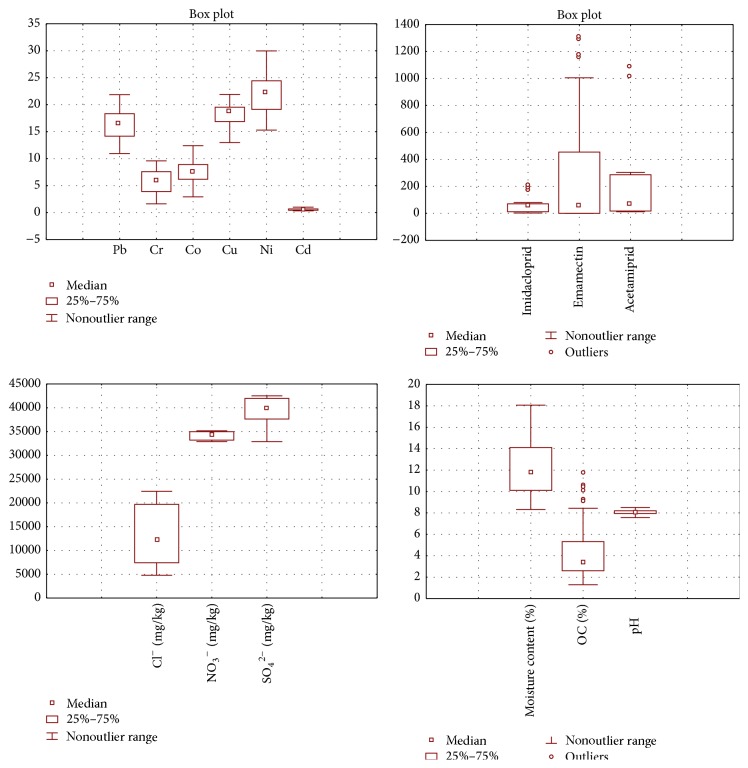
Box and Whisker plots for the distribution of various parameters in soil.

The use of pesticides has contributed significantly towards increased agricultural yield, protection of livestock, and the reduction of vector-transmitted diseases, but their impacts on ecosystems and humans have always been focused on by scientific, regulatory, and policy communities [38–40]. Imidacloprid, acetamiprid, and emamectin were the pesticides that are being applied to the cotton crops of Pakpattan intermittently. That is why these pesticides were included in the study. The data recorded a great variability in the concentrations of imidacloprid that was present at a minimum value of 2.3 *µ*g/g and a maximum value of 205.9 *µ*g/g with a mean value standing at 64.45 *µ*g/g. This large variation in the recorded values reflected the differences in the extent of exposure of soil to the pesticides. Emamectin was present in these samples at mean levels of 296.7 *µ*g/g, while the mean levels for acetamiprid were 264.3 *µ*g/g. The large variation between the mean and median values depicted the nonnormal distribution of the data which was also reflected by high SD, skewness, and kurtosis values. The distribution of these pesticides has also been depicted in Box plots (Figure 2).

The data for the descriptive statistics of selected metals (i.e., Pb, Cr, Co, Cu, Ni, and Cd, Table 2, Figure 2) showed that nickel in these samples was present at highest mean concentration of 22.16 mg/kg. The major source of Ni in these soils may be the Ni plating industry located nearby. Ni was followed by Cu that exhibited a mean concentration of 18.12 mg/kg. The minimum and maximum concentrations recorded for Cu were 12.98 and 21.90 mg/kg, respectively. Copper is required for various biochemical processes in plants; thus it is an essential trace element, but its enhanced concentrations are hazardous for human populations [41].

Lead was another heavy metal that was present in these samples at appreciable maximum concentration of 16.18 mg/kg. Over the past few decades, the vehicular emissions from leaded gasoline have been overcome due to ban on leaded gasoline; thus the source of this lead in soil was traced in various fungicides and pesticides like lead arsenate and so forth that are used at various stages of crop production [2]. Cadmium was the metal that was present at trace level of 0.585 mg/kg. Chromium, another hazardous metal, was found to be present at a mean level of 5.86 mg/kg. Its maximum concentration was recorded to be 9.6 mg/kg which is quite higher than the limit set for agricultural soils. This chromium not only is hazardous for soil biota but may also contaminate the food chain through uptake and accumulation by plants; furthermore depending on the soil conditions like pH, it may become soluble and thus enter the groundwater, the only source of drinking water in the area.

The mean levels recorded for Co were 7.558 mg/kg. The order of the mean concentrations of metals in the soil samples remained: Ni > Cu > Pb > Co > Cr > Cd. The symmetry parameters for the data, that is, standard deviation, skewness, and kurtosis, are also listed in Table 2.

A comparison of mean metal levels with National Environmental Quality Standards (NEQS) is also depicted in Table 2. The average Pb concentration in soil samples was found to be 16.18 mg/kg against the standard value of 25 mg/kg. The estimated value of lead in soil samples was lower than standard value which indicated that soil Pb was below the contamination level and would not pose a significant threat of contaminating the food chain. Moreover, Pb uptake by plants depends upon soil pH; that is, at higher pH, lead becomes immobilized. It is also known that, due to small transfer factor, Pb is not taken up by plants below a concentration of 300 *µ*g/g in soil, and if taken up, it would accumulate in leaves rather than the fruit [42, 43].

The mean level of Cr recorded in soil was 5.86 mg/kg. This value is approximately four times less than standard value of 20 mg/kg (NEQS). Co was present in the soil samples at the mean level of 7.558 mg/kg which is also less than standard value of 10 mg/kg. The mean concentration of Cu in soil samples was found to be 18.12 mg/kg. Ni was present in soil samples at the mean level of 22.16 mg/kg slightly higher than the recommended standard value of 20 mg/kg.

The mean concentration of Cd was found to be 0.585 mg/kg, a value slightly exceeding the standard value of 0.5 mg/kg. In fact, fertilizers are added regularly to soils to replenish N, K, and P for good crop growth. These compounds contain trace amounts of Cd as impurities, which, after continued application, are significantly increased in the soil [44]. Overall, it was concluded that Pb, Cr, Cu, and Co were present in tolerable range in the soil samples but Ni and Cd surpassed the standard value but not to greater extents. Thus although the selected metals do not pose any threat to the environment at their present levels, but with the passage of time, the unwise use of agricultural activities may lead to a large buildup of metals in the soil that may endanger the environment as well as human health.

### 3.2. Correlation Coefficient Matrices of Various Parameters in the Soil

Table 3 depicted the correlation coefficient matrix for the studied physicochemical parameters, pesticides, and metal levels in the soil samples collected from the agricultural fields. The *r*-values were found to be significant at ±0.231 (*p* < 0.05). The data was characterized by both negative and positive correlations between various pairs of parameters. The data was nonnormally distributed and also the anthropogenic activities led to a change in the natural concentration of some of the parameters. Thus all the observed correlations were not high. For the physicochemical parameters, the most significant positive correlation was observed between electrical conductivity and chloride levels with an *r*-value 0.292, evidencing that, in about 29.2% cases, the electrical conductivity of soil was observed to be increased with an increase in chloride content and vice versa. Similarly, a strong positive correlation was observed between pH and sulphate with *r*-values of 0.235. Organic carbon content of soil was found to be positively correlated with nitrate levels but this correlation was not significant.

The correlation coefficient matrix for the levels of selected metals in the collected soil samples depicted the strongest positive correlation between Cr and Ni at an *r*-value of 0.460, thereby showing that in 46.0% cases the concentration of Cr went in parallel with concentration of Ni, thus sharing a similar origin in the soil matrix. The next strongest positive correlation was observed between Ni and Cu pair (0.431). The other metal pair that was significantly positively correlated included Cr-Co (*r*-value 0.343). Cr, Ni, and Cu were found to be strongly negatively correlated with Cd (*r*-values of −0.407, −0.430, and −0.375, resp.) depicting an increase in concentration of one metal with a decrease in concentration of other metals.

No significant, positive, or negative correlation was observed among the selected pesticides depicting that the sources of pesticides in the soil were not the same. This is also due to the fact that different pesticides are applied to the crops at different stages of crop production. Thus they enter the soil at different periods of time. The data corresponding to the correlation coefficient matrix for the physicochemical parameters and the various metals depicted only few significant positive/negative correlations. Ni of the soil exhibited the strongest positive correlation with its conductivity with *r*-value of 0.342. Cu was also significantly positively correlated with the conductivity (*r*-value of 0.286). Cd and electrical conductivity were significantly negatively correlated. Another significant negative correlation was observed between Cr and nitrate level of soil solution. The other correlations were too naïve to be discussed.

The correlation coefficient matrix for the pesticides-to-physicochemical parameters depicted a significant negative correlation between soil organic carbon content and emamectin thereby evidencing the influence of soil organic carbon on the sorption of emamectin. Soil EC values were also found to be negatively correlated with imidacloprid and emamectin.

Numerous metals are known to possess good catalysis ability, and they may affect the behavior of coexisting pesticides in the soil. Thus the most important segment of the present investigation was to study the correlations among the selected pesticides and various metal levels. The data depicted a strong positive correlation between acetamiprid and Cu with *r*-value of 0.239. A strong positive correlation pointed towards an increase in the concentration of acetamiprid with the presence of greater concentration of copper in the soil. On the other hand, imidacloprid in soil was found to be negatively correlated with Ni of soil (*r*-value of −0.317). Similarly, a significant negative correlation was observed between Cr and acetamiprid (*r*-value, −0.305). Thus it was evidenced that Cu stabilizes acetamiprid while Cr and Ni facilitate the degradation of acetamiprid and imidacloprid in the soil [45].

Similar results have been reported for pyrethroid pesticides, whose degradation was shown to be inhibited by the presence of copper ions. Ellis et al. reported that metals like Cu present in soil may react with the sulfhydryl group of enzymes and inhibit their activity. Thus the pesticides become more persistent, thereby leading to an increase in their concentration with time [21, 46].

### 3.3. Cluster Analysis of Various Parameters in the Soil Samples

**Figure 3:**
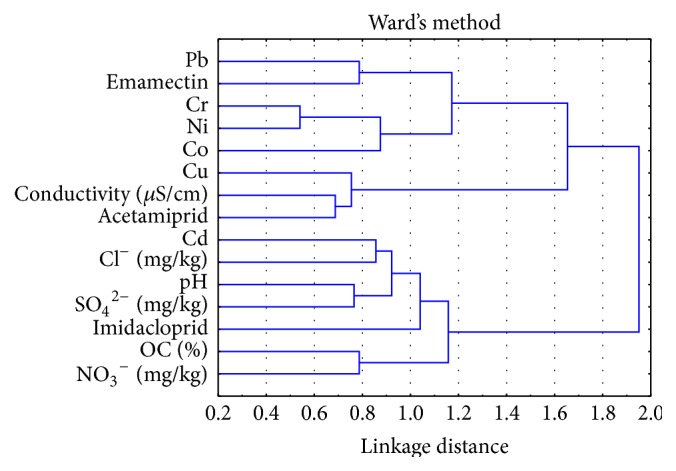
Cluster analysis for the determination of various parameters.

Cluster analysis has proved to be a milestone toward the source apportionment studies of various pollutants like metals and pesticides in the soil and other media [47, 48]. In this analysis (Figure 3), linkage distance is used as the measure of degree of closeness and hence relation among the various parameters [49]. A number of clusters were formed within a significant linkage distance. The strongest cluster was observed between Cr and Ni evidencing that high Cr content of the soil was associated with the enhanced Ni levels. These two metals were further associated with Co to form a second cluster within linkage distance of 1.0. The most important cluster of the study was observed between acetamiprid and conductivity that was further associated with Cu. It evidenced that Cu present in the soil may cause the persistence of acetamiprid whose concentration increases with time. Similarly, emamectin was closely associated with Pd.

Cluster analysis revealed a close association between sulphate and pH, which formed a primary cluster within linkage distance of 0.6. This primary cluster was further associated with Cd and Cl^−^ within linkage distance of 1.0 on one side and with imidacloprid in the soil on the other side. The organic carbon of soil was associated with nitrate within a linkage distance of 0.8 and this factor was further associated with previous cluster.

Overall, the study revealed that frequently used pesticides, that is, imidacloprid, emamectin, and acetamiprid, were present in the soil of cotton fields and were influenced by factors such as soil pH and organic carbon. The study also furnished correlations among pesticides and metals in the soil. It was shown that presence of certain metals such as Ni caused the decreased levels of imidacloprid. Similarly, Cr enhanced the degradation of acetamiprid and Cu caused the persistence of this pesticide in the soil.

## 4. Conclusions

The soil samples collected from the cotton growing area of Pakpattan, Pakistan, were slightly basic in nature. The mean Pb, Cr, Co, and Cu levels were below NEQS while the levels of Ni and Cd were slightly above NEQS. These levels may be tolerated by following precautionary measures in agricultural activities. Among the pesticides, emamectin exhibited a high persistence in the soil that was closely associated with soil Cu content. It was also found that Cu stabilizes acetamiprid while Cr and Ni facilitate the degradation of acetamiprid and imidacloprid. Thus the study provides basis for the risk assessment studies that are quite necessary to manage the contaminated sites in a cost effective manner while preserving the public and ecosystem health. It also provides a management strategy for remediation of contaminated soil; that is, adding traces of Ni and Cr in the soil may help in the degradation of frequently used acetamiprid and imidacloprid.

## Figures and Tables

**Table 1:** Analytical parameters for the estimation of selected metals by AAS.

Sr. number	Metal	Wavelength (nm)	Lamp current (mA)	Slit width (nm)	Fuel flow rate (L/min)	Detection limit (mg/L)
1	Pb	283.3	9.0	1.3	2.2	0.20
2	Cr	359.3	9.0	1.3	2.8	0.05
3	Co	240.7	15	0.2	2.2	0.20
4	Cu	324.8	9.0	1.3	2.2	0.02
5	Ni	232.0	12	0.2	2.2	0.10
6	Cd	228.8	9.0	1.3	2.0	0.005

**Table 2:** Basic statistics parameters for the distribution of selected parameters (mg/kg) in soil samples (*n* = 52).

	Minimum	Maximum	Mean	Median	SD	SE	Variance	Kurtosis	Skewness	NEQS	BC^*∗*^
Pb (mg/kg)	10.93	21.85	16.18	16.49	2.68	0.372	7.184	−0.639	−0.261	25	18.9
Cr (mg/kg)	1.64	9.6	5.86	5.995	2.238	0.31	5.008	−0.902	−0.183	20	50.3
Co (mg/kg)	2.93	12.4	7.558	7.595	2.094	0.29	4.386	−0.291	0.077	10	0.392
Cu (mg/kg)	12.98	21.90	18.12	18.56	2.141	0.297	4.584	−0.332	−0.516	—	27.6
Ni (mg/kg)	15.3	29.97	22.16	22.25	3.666	0.508	13.44	−0.572	0.118	20	0.135
Cd (mg/kg)	0.347	1.019	0.585	0.538	0.175	0.024	0.031	0.104	0.961	0.5	0.14
Bulk density (Mgm^−3^)	1.530	1.610	1.568	1.570	0.028	0.005	0.001	−1.333	−0.003	—	—
OC (%)	1.291	11.39	4.616	3.390	2.894	0.401	8.376	−0.248	1.074	—	4.5
pH	7.6	8.5	8.1	8.055	0.2	0.028	0.04	−0.07	−0.206	—	7.2
Conductivity (*µ*S/cm)	980	1887	1412	1600.5	317.4	44.02	1.008 × 10^5^	−1.777	−0.076	—	108
Cl^−^ (mg/kg)	4808	22438	12909	12338	6145.6	852.2	3.777 × 10^7^	−1.288	0.271	—	987
NO_3_^−^ (mg/kg)	2879	94434	35009	34470	10852	1505	1.178 × 10^8^	18.54	2.807	—	843
SO_4_^2−^ (mg/kg)	4.009 × 10^3^	9.297 × 10^4^	4.710 × 10^4^	4.0 × 10^4^	2.159 × 10^4^	2.993 × 10^3^	4.660 × 10^8^	1.028	1.447	—	419
Imidacloprid (*µ*g/g)	2.3	205.9	64.45	55.6	62.12	8.613	3857	0.36	1.229	—	—
Emamectin (*µ*g/g)	0.001	1305	296.7	59.9	431.2	59.8	1.859 × 10^5^	0.414	1.351	—	—
Acetamiprid	11.40	1258.8	264.3	70	411.83	57.11	169600	0.968	1.635	—	—

^*∗*^BC = background levels (mean of three).

**Table 3:** Correlation coefficient matrix^a^ for various parameters of soil samples (*n* = 52).

	Pb	Cr	Co	Cu	Ni	Cd	OC^b^	pH	EC^c^	Cl^−^	NO_3_^−^	SO_4_^2−^	Im^d^	Em^e^	Ac^f^
Pb	1.000														
Cr	0.179	1.000													
Co	0.019	**0.343**	1.000												
Cu	0.018	0.132	−0.002	1.000											
Ni	0.199	**0.460**	0.074	**0.431**	1.000										
Cd	−0.154	**−0.407**	−0.045	**−0.375**	**−0.430**	1.000									
OC (%)	0.124	0.083	0.092	−0.136	−0.039	−0.075	1.000								
pH	0.080	−0.162	0.164	−0.124	−0.076	0.118	0.187	1.000							
EC (*µ*S/Cm)	−0.009	−0.045	−0.047	**0.286**	**0.342**	**−0.245**	0.137	0.059	1.000						
Cl^−^ (mg/kg)	−0.163	**−0.255**	−0.140	−0.079	0.034	0.144	0.083	0.058	**0.292**	1.000					
NO_3_^−^ (mg/kg)	0.011	0.018	−0.221	−0.065	0.051	−0.076	0.213	0.014	0.153	0.087	1.000				
SO_4_^2−^ (mg/kg)	−0.080	−0.149	−0.201	−0.096	−0.057	0.146	0.004	**0.235**	−0.156	0.212	0.177	1.000			
Im^*∗*^	0.086	−0.021	0.012	0.017	**−0.317**	0.021	0.100	0.175	−0.314	−0.122	−0.103	0.052	1.000		
Em^*∗*^	0.213	−0.071	0.161	0.009	−0.012	0.015	**−0.239**	0.084	−0.265	−0.163	−0.183	0.085	−0.086	1.000	
Ac^*∗*^	−0.119	**−0.305**	−0.143	**0.239**	−0.102	0.163	−0.077	0.099	0.313	0.038	0.007	−0.085	0.050	−0.174	1.000

^a^ *r*-values are significant at 0.231 at *p* < 0.05.

^b^OC = organic carbon, ^c^EC = electrical conductivity, ^d^Im = imidacloprid, ^e^Em = emamectin, ^f^Ac = acetamiprid.

^*∗*^Concentration in *µ*g/g.
